# Research on Components Assembly Platform of Biological Sequences Alignment Algorithm

**DOI:** 10.3389/fgene.2020.630923

**Published:** 2021-01-21

**Authors:** Haihe Shi, Gang Wu, Xuchu Zhang, Jun Wang, Haipeng Shi, Shenghua Xu

**Affiliations:** ^1^School of Computer and Information Engineering, Jiangxi Normal University, Nanchang, China; ^2^School of Information Management, Jiangxi University of Finance and Economics, Nanchang, China; ^3^School of Software, Jiangxi Normal University, Nanchang, China

**Keywords:** biological sequence alignment algorithm, component, component model, component assembly platform, B/S architecture

## Abstract

After years of development, the complexity of the biological sequence alignment algorithm is gradually increasing, and the lack of high abstract level domain research leads to the complexity of its algorithm development and improvement. By applying the idea of software components to the design and development of algorithms, the development efficiency and reliability of biological sequence alignment algorithms can be effectively improved. The component assembly platform applies related assembly technology, which simplifies the operation difficulty of component assembly and facilitates the maintenance and optimization of the algorithm. At the same time, a friendly visual interface is used to intuitively complete the assembly of algorithm components, and an executable sequence alignment algorithm program is obtained, which can directly carry out alignment computing.

## Introduction

Bioinformatics is an interdisciplinary subject involving life sciences, mathematics, and computer science. Its main research work lies in the acquisition, processing, and storage of biological information, and further includes distribution, analysis, and interpretation. Its research methods are to use various technologies and tools of computer, biology and mathematics to mine and understand the biological significance contained in the massive data (Wang et al., 2015; Liu, 2018). After years of development, bioinformatics has shaped big data of biological information. As a basic method of mining biological sequence information, sequence alignment algorithms have received extensive attention from researchers in recent years.

Sequence alignment algorithms can be divided into pairwise sequence alignment algorithms and multiple-sequence alignment algorithms(Zhan et al., 2019, 2020). The most classic solution of the pairwise sequence alignment algorithm is the dynamic programming algorithm, and the multiple-sequence alignment algorithm is due to its NP completeness (Wang and Jiang, 1994), the current research is dedicated to finding the best approximate solution, but there is a lack of research on the level of algorithm domain. In recent years, the complexity and development difficulty of the newly proposed sequence alignment algorithm program have been increasing, and the efficiency of algorithm development and maintenance cannot be guaranteed. The idea of Component-Based Software Development (CBSD) (Yin, 2017) is viewed as an effective means to solve the “software crisis.” It is also one of the current development trends of software development. Its greatest advantage is that it can reuse the existing development results and improve software development efficiency. Algorithm is the core of software, which embodies the wisdom of software developers. The development efficiency and running efficiency of the algorithm have a crucial impact on the final quality of software. Therefore, the development idea of CBSD can be applied to algorithm development to further improve the development activities of algorithm programs.

Don Batory proposed an algorithmic component development method, connecting the feature model, grammar, and proposition formula to achieve the purpose of defining arbitrary constraints and using satisfiability solvers to debug feature models. In addition, a logical truth maintenance system is introduced to propagate the constraint characteristics of feature selection. Finally, based on these theoretical foundations, a product line development tool set that supports feature modularization and its combination is developed, and the combination development of graph algorithm is described (Batory, 2005).

Through in-depth study, we found that the first step of component-based algorithm development is to complete the domain analysis of algorithm family in a certain domain, and obtain a domain feature model that can guide component design and implementation. Next step is the structural design and interaction design of the components according to the requirements shown in the feature model. Finally it is to implement models using a suitable development language and provide corresponding component assembly services. Under the guidance of generative programming (Czarnecki and Eisenecker, 2000), FODM (Zhang and Mei, 2003) domain modeling method and PAR (Xue, 1993, 1997, 1998, 2016; Wang and Xue, 2009; Xue et al., 2018), domain modeling activities, component design activities and component implementation activities for common sequence alignment algorithms are almost done by our research team. Based on the existing results, the paper presents the assembly platform of sequence alignment algorithm components. The platform mainly provides the assembly services for the developed algorithm components, which greatly improves the automation of the algorithm component assembly, and further reduces the complexity of the algorithm development.

## Platform Construction

### Preliminaries

#### Software Reuse

With the development of computer technology, its influence in human society is gradually improved. While the complexity and security of software are becoming increasingly prominent. Researchers are difficult to grasp the efficiency, cost, quality and future maintenance of software development. As early as 1968, the North Atlantic Treaty Organization (NATO) has put forward the definition of software crisis. And then the research of software engineering (Wang et al., 2018) also develops rapidly. Software reuse (Zhang and Mei, 2003, 2014; Zhang et al., 2005; Barros-Justo et al., 2019; Feng et al., 2019) is considered to be a feasible technology to improve the level of software industrial production and effectively solve the software crisis.

The idea of software reuse is to reuse the existing software in accordance with the specifications in the development process. When developing other systems in the same field, it is not necessary to develop from scratch, but on the basis of reusable resources to carry out efficient reuse development. In this process, abstraction is the basic element (Zhu, 2017), and efficient reuse cannot lack high-abstraction modeling of related reuse fields. The scope of reusable resources covers various forms of products, including software design documents, domain models, software patterns, code components, software architecture, software implementation documents, application generators and so on.

#### Component Technology

Component assembly technology (Zhang, 2018; Wu, 2019) is the core part of realizing CBSD. After completing a series of component design and development work, the final goal of CBSD is to assemble the components. From the current research (Xu et al., 2006; Chen et al., 2012; Zhen et al., 2014), the technology has achieved some research results.

The component assembly forms mainly include black box assembly, white box assembly and gray box assembly. The main difference is whether the components need to be modified before assembly. Black box assembly is the most suitable assembly method for component encapsulation, but it also reduces the adaptability of components. White box assembly emphasizes the adaptability of components. The assembly is flexible and can achieve greater composability. However, due to too many implementation details exposed, the ease of use of components will be reduced, and improper modifications will occur, so that the final assembly cannot achieve the expected. Gray box assembly is the most widely used assembly method currently. It combines black box assembly and white box assembly and can be adapted to a variety of application scenarios.

The difference between sequence alignment algorithm component and software component is that the former often has higher coupling degree, and the algorithm component often needs to be modified to adapt to the relevant application scenarios. However, the idea of sequence alignment algorithm is complex. If the assembler does not have a good understanding of the algorithm, new errors will be made when modifying it. Therefore, before assembling algorithm components, it is necessary to conduct a detailed domain analysis, formally describe the algorithm component, and form a structure framework to guide the algorithm component assembly. Finally, the gray box assembly of sequence alignment algorithm components is completed under the guidance of domain model, formal specification and algorithm framework.

### Platform Design

#### Requirement Analysis

The goal of CBSD is that the software system can be automatically generated from a series of software components according to the system requirements supported by the generator. The purpose of developing the component assembly platform for sequence alignment algorithm is also to reduce the manual assembly workload as much as possible and improve the automation level of the whole component system.

**Figure 1:**
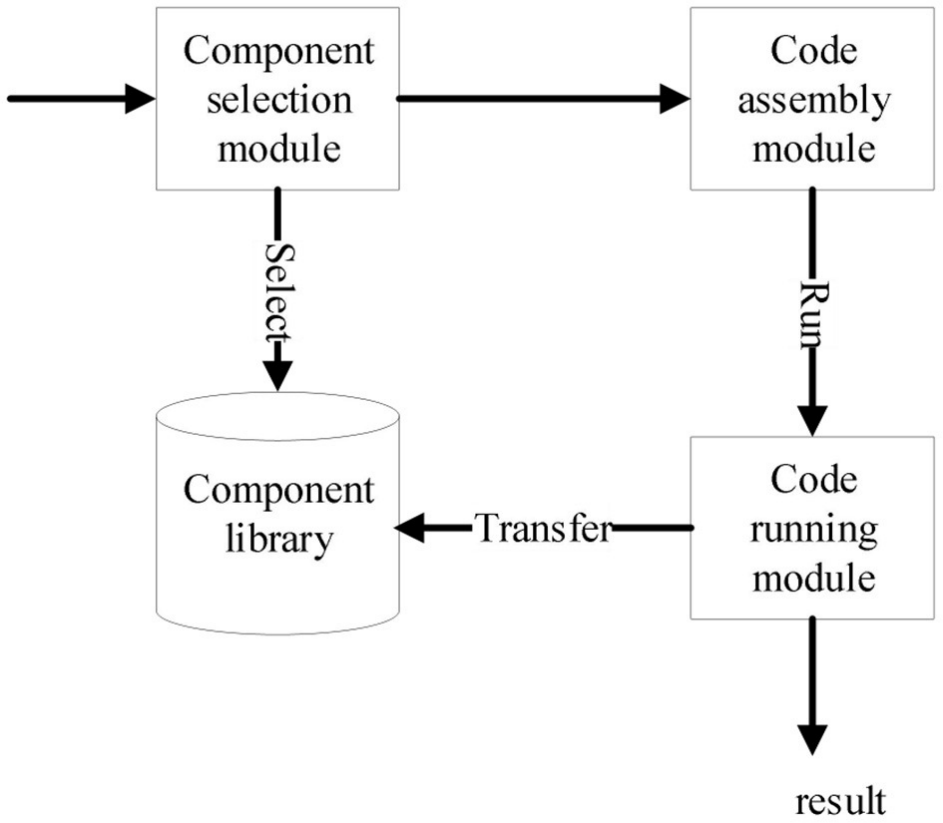
Interactions among component assembly modules.

The platform mainly includes component transformation, component assembly and code running. By means of C++ program generation system of PAR, the component transformation module can transform Apla components into C++ components, see details in Xue et al. (2018). The component assembly function and code running function are composed of four modules, i.e., component library, component selection, code assembly, and code running. The interactive relationship among the modules is shown in Figure 1.

Component library module includes two parts, one is the source code of algorithm components that have been transformed and stored in the files, and the other is the Apla component assembly code that needs to be manually developed or modified in the database. In addition, the component library module also plays a management role, such as adding, deleting, modifying, and checking components, supporting further component expansion and modification in the future.

Component selection module reads the components in the component library and displays them on the platform interface according to their required and optional features and the type characteristics of their affiliation. After selecting the components, the validity of component composition is checked. According to the multi-choice one or multi-choice relationship of feature dimension and the dependency relationship between components, the component composition is constrained accordingly to prevent illegal combination from the subsequent process.

After completing the component selection, code assembly module obtains the required Apla assembly code from the database, and the user can make appropriate modifications to correctly call the selected component in the component library. After the assembly code is developed, the Apla code is converted into the corresponding C++ code through the transformation system of PAR platform. Finally, the assembly and compilation of executable codes is completed in the sever of platform to generate executable algorithmic programs.

After the user inputs the alignment data, the code running module executes the corresponding executable alignment algorithm program. It enables the user to directly perform sequence alignment operations on the platform, and displays the final algorithm running results on the page.

#### System Design

The platform is developed using B/S architecture following J2EE specifications, the Java language is used, and the Spring Boot (Wang et al., 2016) and MyBatis (Rong, 2015) as well as Thymeleaf framework, which are currently popular in web development, are adopted, and the stability of their architecture has been tested by practical applications.

**Figure 2:**
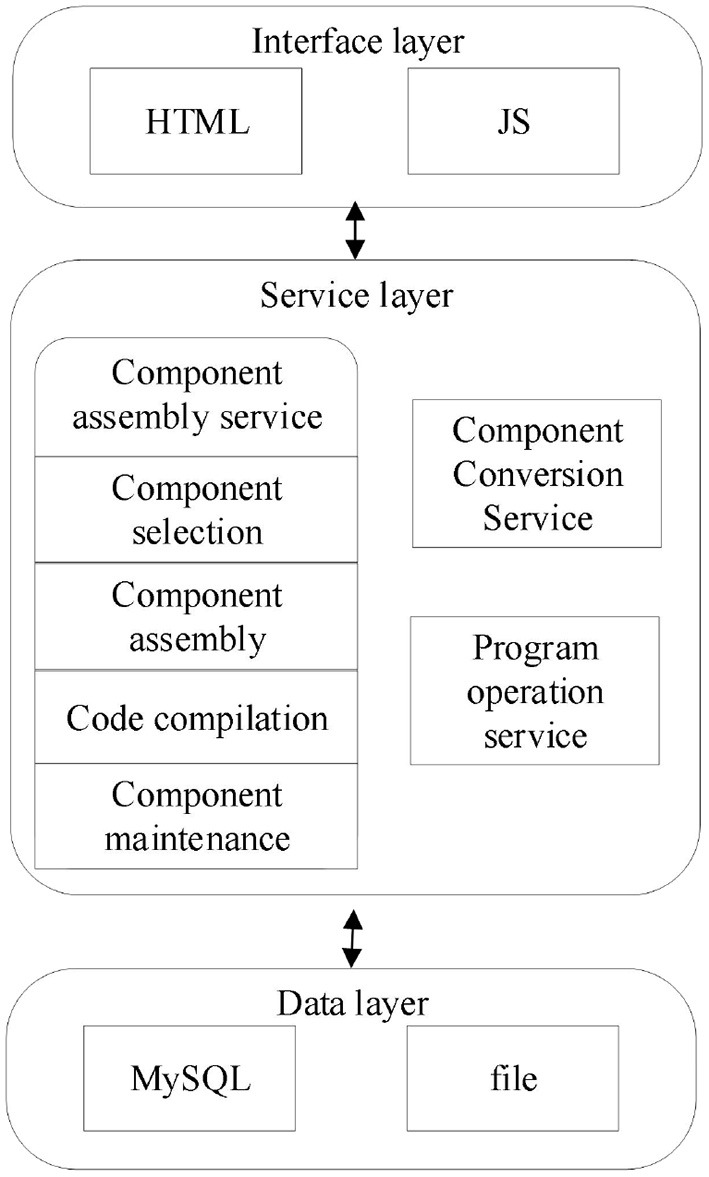
Overall platform architecture.

The overall architecture of the assembly platform is shown in Figure 2, including the data layer, service layer, and interface layer. The data layer mainly uses MySQL and files to store the Apla program and component source code required by the platform. The service layer mainly includes component assembly service, component transformation service, and program run service. The application layer mainly uses HTML to display the platform, uses JS to implement the relevant interaction logic.

**Figure 3:**
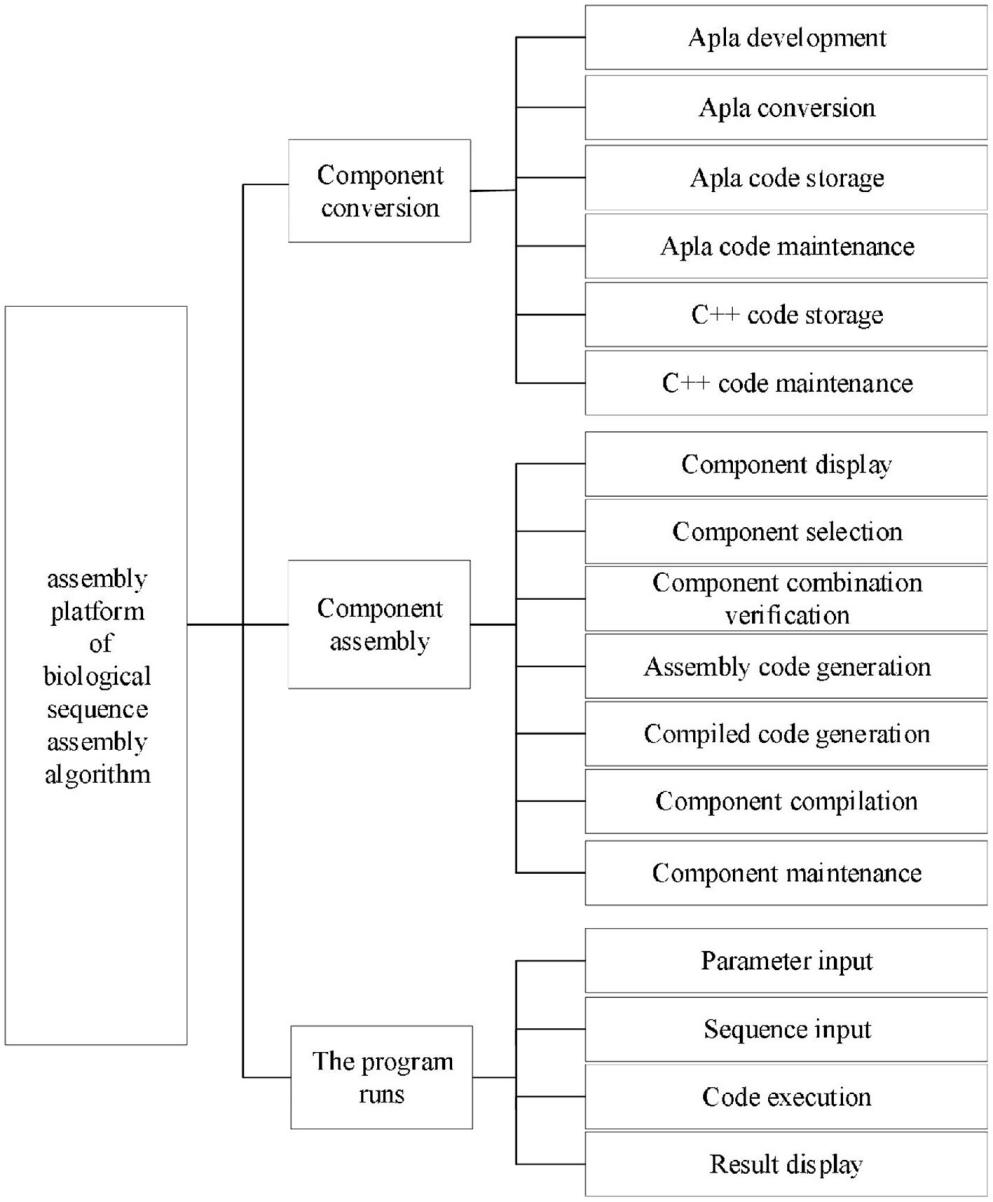
Platform functional architecture.

According to the requirement analysis, the functional architecture of the platform is shown in Figure 3. Component transformation module includes the functions of Apla development, Apla transformation, Apla code storage and maintenance, and C++ code storage and maintenance. As the core of the platform, component assembly module consists of the functions of component display, component selection, component combination verification, assembly code generation, compiling code generation, component compilation and component maintenance. Program running module is composed of the functions of sequence input, parameter input, code execution and result display.

#### Detailed Platform Design

**Figure 4:**
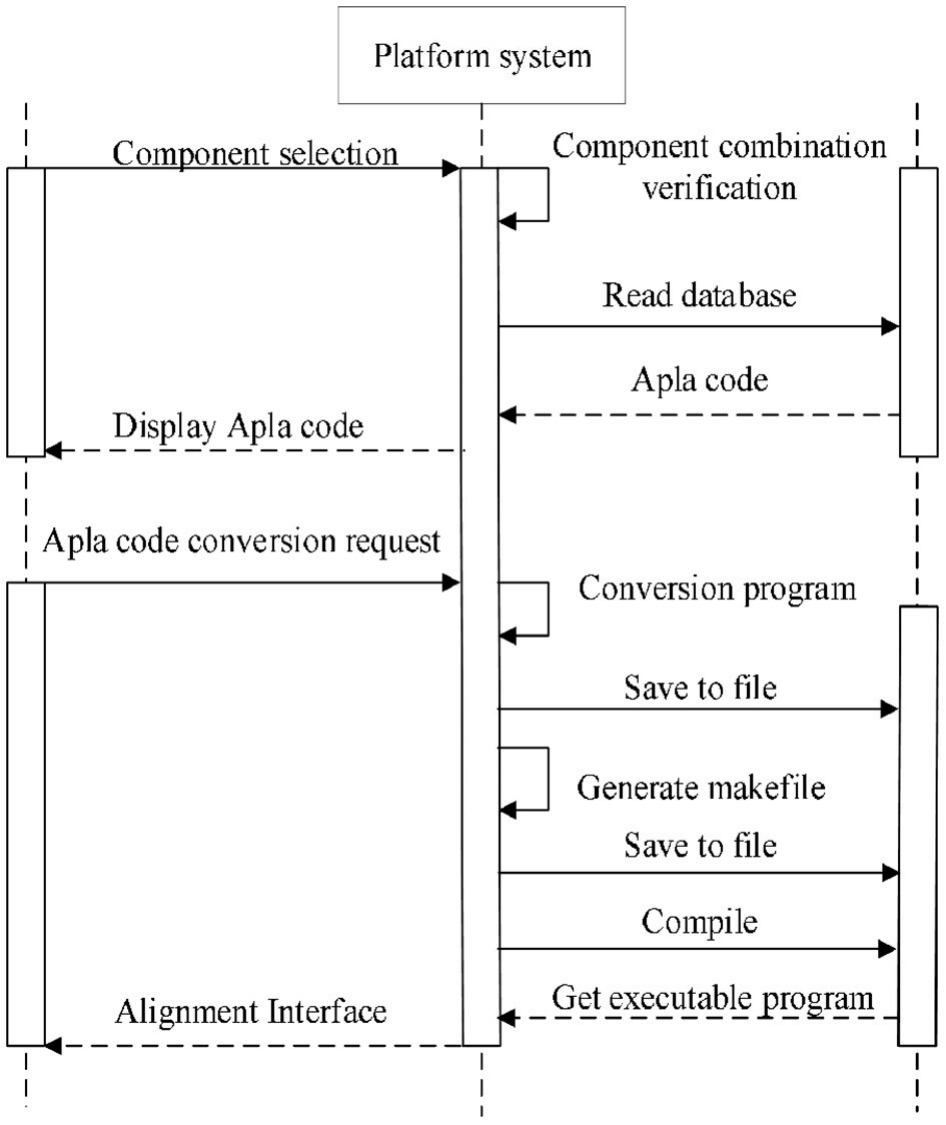
Component assembly process.

Through system function requirement analysis, overall architecture design, and functional architecture design, the sequence alignment algorithm component assembly platform is outlined. Next is to give a detailed design of the platform system based on the operating sequence of each module. The platform mainly includes component conversion process, component selection process, component assembly process and algorithm execution process. This section mainly describes the process of component assembly, as shown in Figure 4.

### Analysis of Key Platform Algorithms

**Figure 5:**
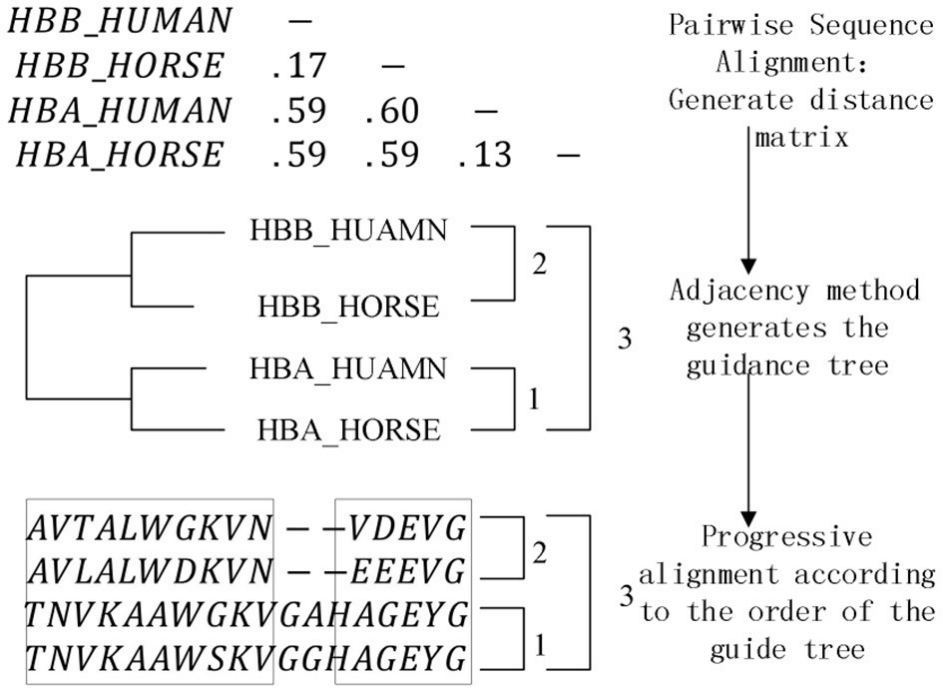
Steps of ClustalW.

The pairwise sequence alignment algorithm and heuristic multiple sequence alignment algorithm based on dynamic programming have been implemented in the platform, and the most critical one is the progressive multiple sequence alignment algorithm based on phylogenetic tree. The most classic ClustalW (Thompson et al., 1994) algorithm in the algorithm thought is implemented in 1994 by Thompson and Higgins. Its operation steps are described as follows, and the algorithm diagram is shown in Figure 5.

1. Pairwise sequence alignment. The sequence group is aligned between two pairs, and the distance matrix is established by the pairwise sequence alignment score to indicate the distance between the sequences.
2. Generate a phylogenetic tree. Using the information in the distance matrix, a phylogenetic tree is established through the corresponding clustering algorithm to guide the subsequent multiple sequence alignment operations.
3. Progressive alignment. The previous operation has generated a guide tree, and the last step is to gradually complete the alignment of all sequences in the form of keeping gaps, starting from the close evolutionary relationship according to the alignment sequence reflected by the guide tree.

The components involved in the algorithm are sequence validity check component, pairwise sequence alignment component, distance matrix component, phylogenetic tree component, progressive alignment component, and alignment result output component. Since the distance matrix component and the pairwise sequence alignment component are highly coupled, the pairwise sequence alignment component is designed as a generic operation parameter of the distance matrix, and the corresponding distance matrix can be generated by instantiating different pairwise sequence alignment algorithms. The phylogenetic tree component also includes a clustering algorithm selection sub-component, which is also designed as a generic operation parameter. The commonly used instantiation algorithms are the NJ algorithm (Saitou and Nei, 1987) and the UPGMA algorithm (Zhang et al., 2018). The objective function (Carrillo and Lipman, 1988; Notredame et al., 1998) is also designed as a generic operation parameter while performing a progressive alignment, here we aims to expand the scope of algorithm components assembly.

## Assembly Example

We will carry out an example of the assembly for the progressive alignment algorithm based on phylogenetic tree to demonstrate how the modules of the platform work together and how they interact with each other.

**Figure 6:**
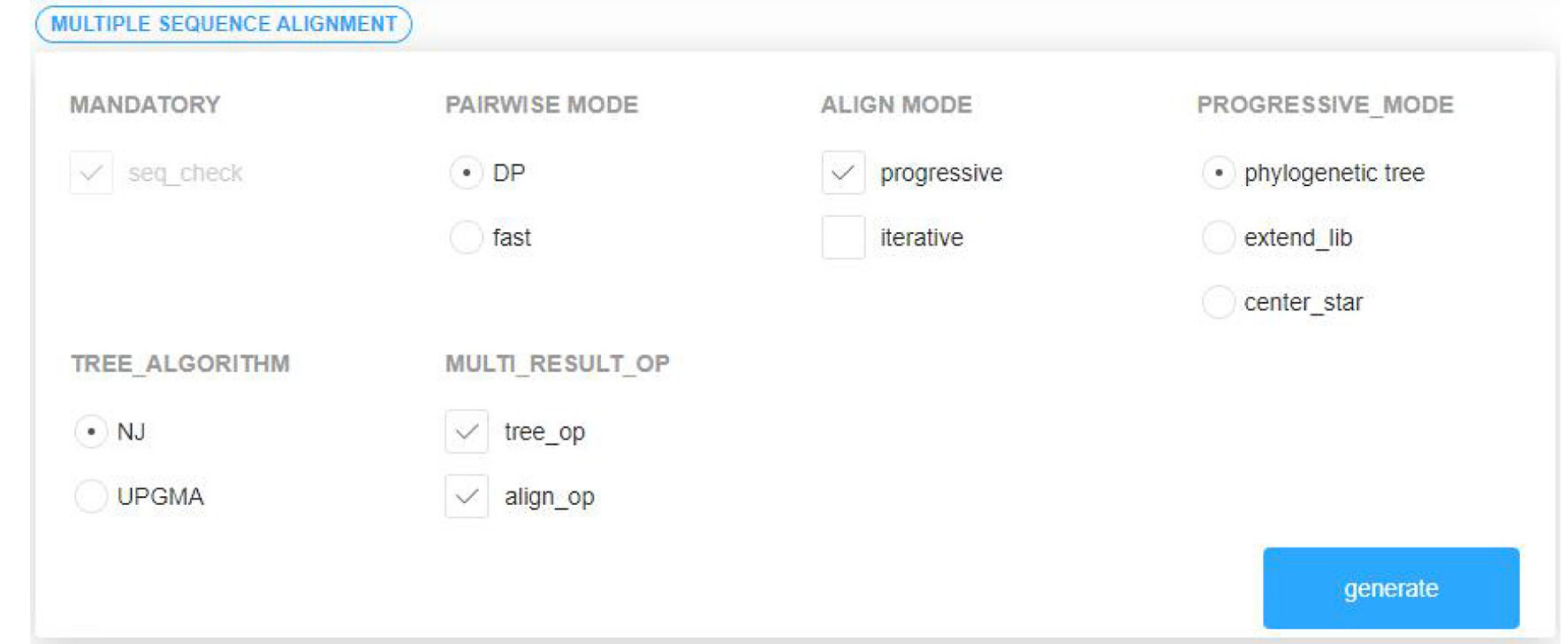
Component selection interface.

**Figure 7:**
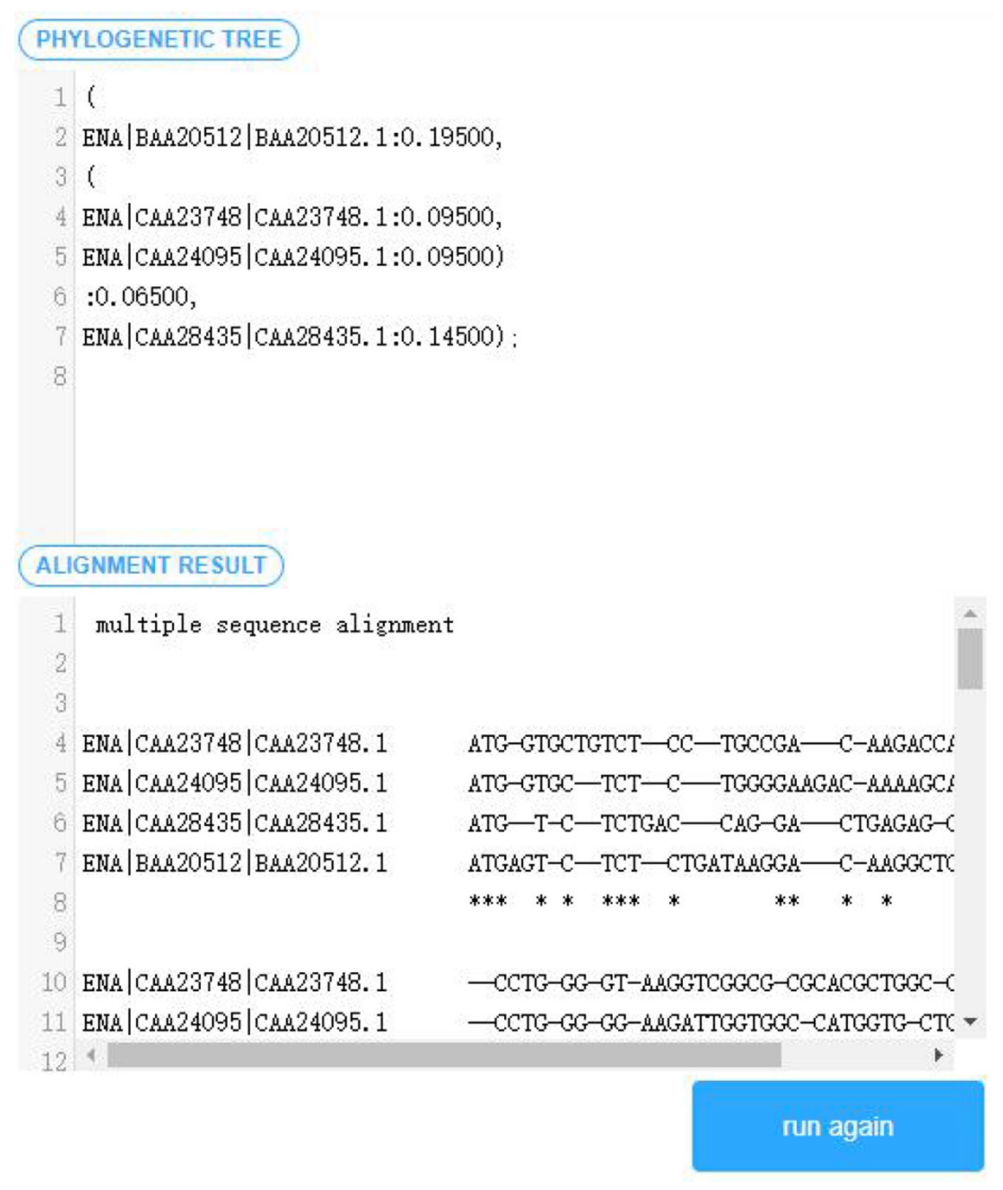
Alignment result interface.

1. Transform Apla components except those for assembling. The transformation system of PAR platform is used to convert the developed Apla components into C++ components and store them to the platform's local files.
2. Visually select several existing components satisfying the composable constraints according to the established domain feature model and component interaction model. The components are grouped by the required or optional attribute. In order to prevent the selection of illegal combinations from the subsequent assembly, the distinction between multi-choice-one or multi-choice-multi is carried out in the optional components group. The component selection interface is shown in Figure 6.
3. Based on the interaction relationship between the components, read the corresponding Apla assembly code in the database and display it on the page following the selection of component combination. The user can check and modify the component assembly code, and then submit an Apla conversion request and store the converted C++ assembly code as the local file.
4. After all the Apla component conversion and assembly code conversion, the *makefile* script file for compilation is generated automatically, and is executed to compile and link the C++ components. The parameter input interface of sequence the Presentation 1 for details.
5. After the user inputs the sequence data, and the replacement matrix as well as the penalty model required by the multiple sequence alignment, the algorithm program generated will be executed, and the alignment output displayed in the user interface. As shown in Figure 7.

## Summary

With the development of CBSD, component-based development technology has been verified in many practical applications. It can exactly improve the development efficiency and maintenance of software. In this paper, the component development technology is applied to the development of biological sequence alignment algorithms. Under the guidance of domain modeling, generative programming and PAR, the formal transformation of sequence alignment algorithm components is carried out, and a B/S-based visual assembly platform for the gray box assembly of algorithm components is constructed. On top of our previous study results, the components required by the sequence alignment algorithm are classified and displayed, and the corresponding combination constraints are designed and implemented. After the legal component combination is selected, the assembly code can be modified and compiled to form an executable algorithm program. In addition, the algorithm can run directly on the platform to facilitate users to conveniently conduct sequence alignment studies.

Next, we will release out codes in GitHub. Future work also includes the improvement of the biological sequence alignment algorithm component assembly platform from the following aspects.

1. The algorithm components of this platform will be further expanded to enlarge the scope of algorithms generated from component assembly.
2. The combination constraints in the platform have not been explicitly implemented. We will restrict the combination constraints of algorithm components to XML files, and shape the corresponding combination constraint documents to make it easier for users to assemble.
3. With the richer component library, the algorithm component library needs to have an efficient component search function. Recent years, the recommendation algorithm based on artificial intelligence has developed rapidly. The feasibility of introducing this technology into the platform to improve the ease and automation level of algorithm assembly platform will be carefully studied.

## Data Availability Statement

The original contributions presented in the study are included in the article/Supplementary Material, further inquiries can be directed to the corresponding author/s.

## Author Contributions

HS instructed the whole research work and revised the paper. GW, XZ, and JW did the codes work and the experiments. HS did the experiments. SX proofread the full text. All authors read and approved the final manuscript and were agreed to be accountable for all aspects of the work.

## Conflict of Interest

The authors declare that the research was conducted in the absence of any commercial or financial relationships that could be construed as a potential conflict of interest.
