# Supplementary material for: Research on Components Assembly Platform of Biological Sequences Alignment Algorithm
Source: Front Genet. 2021 Jan 21;11:630923. doi: 10.3389/fgene.2020.630923 (PMC7859483; doi:10.3389/fgene.2020.630923)
Supplement: Supplementary file 1 [file Presentation_1.pdf]

The generated make-file is shown below.

```
CC=g++

CFLAGS=-c

TARGET=msa

SOURCE=submatrix.cpp multipleSequence.cpp readSeq.cpp
mode.cpp msaCheck.cpp resultOp.cpp \

NJTree.cpp phy_tree.cpp unrootedTree.cpp
dist_matrix.cpp \ AlignSteps.cpp MSA.cpp
ProfileAlign.cpp \

TARGETS = $(patsubst %cpp,%o,$(SOURCE))

$(TARGET):$(TARGETS) main.o

 $(CC) $^ -o $(TARGET)

$(TARGETS):$(SOURCE)

 $(CC) $(CFLAGS) $^

main:main.cpp

 $(CC) $(CFLAGS) main.cpp

.PHONY:clean

clean:

 del *.o *.aln *.fa.dnd *.fa
```
